# POSITIVE AFFECT AND SOCIAL SUPPORT SATISFACTION AMONG NEWLY ADMITTED LONG-TERM CARE RESIDENTS

**DOI:** 10.1093/geroni/igac059.2856

**Published:** 2022-12-20

**Authors:** Darby Simon, Benjamin Mast, Emilee Ertle, Suzanne Meeks

**Affiliations:** University of Louisville, Louisville, Kentucky, United States; University of Louisville, Louisville, Kentucky, United States; University of Louisville, Louisville, Kentucky, United States; University of Louisville, Louisville, Kentucky, United States

## Abstract

Social support serves as a protective factor against depression and other negative outcomes among older adults. Satisfaction with social support among long-term care residents has not received as much attention, and the experience of support among newly admitted long-term care residents has not been investigated. The current study examines what variables influence a long-term care resident’s satisfaction with their social support. This sample consists of 48 individuals newly admitted into long-term nursing home care. This study used the Social Support Questionnaire (SSQ6) to gather information on social support network size and overall satisfaction of perceived social support. Correlations were estimated between social support satisfaction, social support network size, and psychosocial factors including depression (PHQ-9), anxiety (RAID), religious involvement, cognitive status (BIMS), positive and negative affect (PANAS). Satisfaction with support was significantly correlated with social network size (r = .328, n = 48, p < .05) and positive affect (r = .437, n = 24, p < .05). In a regression model with support satisfaction as the dependent variable, both social network size and positive affect contributed significantly to model R2 F(2,21) = 2.441, p = .049 R2 = .250. Satisfaction with support depends upon the number of supporters available, but also on levels of positive affect.

